# Supplementary material for: Hippocampal CA1 Somatostatin Interneurons Originate in the Embryonic MGE/POA
Source: Stem Cell Reports. 2019 Oct 17;13(5):793–802. doi: 10.1016/j.stemcr.2019.09.008 (PMC6895756; doi:10.1016/j.stemcr.2019.09.008)
Supplement: Document S1. Figure S1 [file mmc1.pdf]

Stem Cell Reports, Volume *13*

Supplemental Information

Hippocampal CA1 Somatostatin Interneurons Originate in the Embryonic MGE/POA

Zeinab Asgarian, Lorenza Magno, Niki Ktena, Kenneth D. Harris, and Nicoletta Kessaris

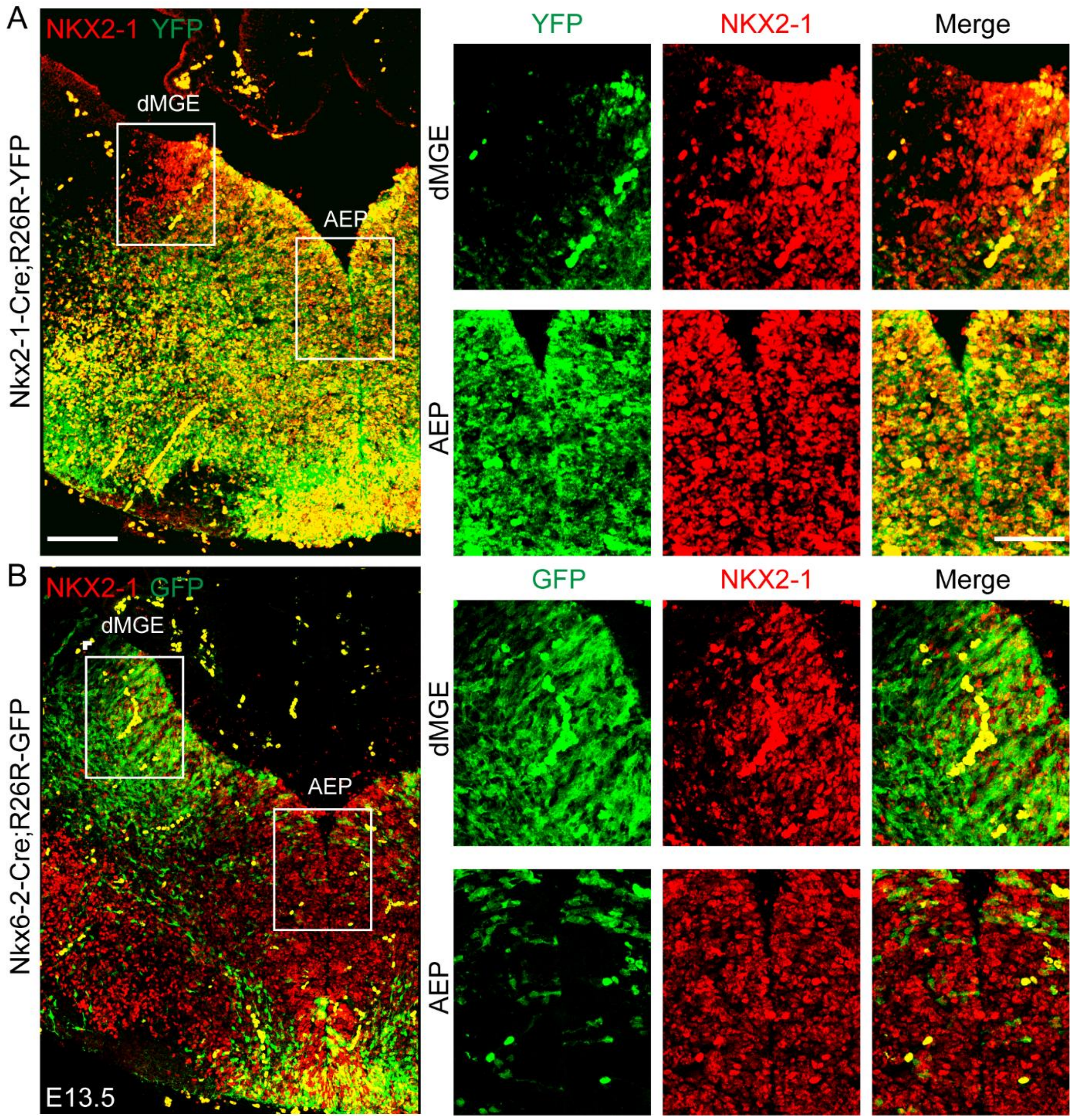

Figure S1 (Related to Figure 1):

Expression of NKX2-1 protein in the MGE and AEP in Nkx2-1-Cre;YFP and Nkx6-2-Cre;GFP mice at E13.5

A) NKX2-1 expression in Nkx2-1-Cre;YFP at E13.5. Expression of endogenous NKX2-1 and activation of YFP expression overlap in the MGE and AEP but not in the dMGE. The dMGE and AEP are shown at higher magnification.

B) NKX2-1 expression in Nkx6-2-Cre;GFP at E13.5. Activation of GFP expression is observed in the dMGE where NKX2-1 protein can be detected but not in the AEP. dMGE, dorsal medial ganglionic eminence; AEP, anterior entopeduncular area. Scale bars: a, b: 100 μm (left), 60 μm (right).
