# Supplementary material for: Hippocampal CA1 Somatostatin Interneurons Originate in the Embryonic MGE/POA
Source: Stem Cell Reports. 2019 Oct 17;13(5):793–802. doi: 10.1016/j.stemcr.2019.09.008 (PMC6895756; doi:10.1016/j.stemcr.2019.09.008)
Supplement: Document S2. Article plus Supplemental Information [file mmc2.pdf]

Stem Cell Reports
Report
OPEN ACCESS

Hippocampal CA1 Somatostatin Interneurons Originate in the Embryonic MGE/POA

Zeinab Asgarian,[1,2] Lorenza Magno,[1,2] Niki Ktena,[1] Kenneth D. Harris,[3] and Nicoletta Kessaris[1,2,*]
[1]Wolfson Institute for Biomedical Research, University College London, Gower Street, London WC1E 6BT, UK
[2]Department of Cell and Developmental Biology, University College London, Gower Street, London WC1E 6BT, UK
[3]UCL Institute of Neurology at the Cruciform Building and Department of Neuroscience, Physiology, and Pharmacology, University College London, Gower Street, London WC1E 6BT, UK
*Correspondence: n.kessaris@ucl.ac.uk
https://doi.org/10.1016/j.stemcr.2019.09.008

SUMMARY

Oriens lacunosum-moleculare (O-LM) interneurons constitute 40% of hippocampal interneurons expressing Somatostatin (SST). Recent evidence has indicated a dual origin for these cells in the medial and caudal ganglionic eminences (MGE and CGE), with expression of *Htr3a* as a distinguishing factor. This is strikingly different from cortical SST interneurons that have a single origin within the MGE/preoptic area (POA). We reassessed the origin of hippocampal SST interneurons using a range of genetic lineage-tracing mice combined with single-cell transcriptomic analysis. We find a common origin for all hippocampal SST interneurons in NKX2-1-expressing progenitors of the telencephalic neuroepithelium and an MGE/POA-like transcriptomic signature for all SST clusters. This suggests that functional heterogeneity within the SST CA1 population cannot be attributed to a differential MGE/CGE genetic origin.

INTRODUCTION

The medial and caudal ganglionic eminences (MGE and CGE) are the two major germinal zones generating GABAergic interneurons for the neocortex (Wonders and Anderson, 2006). The prime distinguishing factor between the MGE and CGE progenitor pools is the expression of NKX2-1 within the neuroepithelium. Genetic lineage tracing in transgenic mice largely confirmed findings from classical embryology studies showing that the MGE generates Somatostatin (SST)-expressing and Parvalbumin (PV)-expressing interneurons (Fogarty et al., 2007; Xu et al., 2008), whereas most Calretinin (CR), Neuropeptide Y (NPY), all Reelin (RLN)$^{+}$ SST^{-}, and all Vasoactive intestinal peptide cells originate in the CGE (Miyoshi et al., 2010; Rubin et al., 2010). The preoptic area (POA) is an additional small source of cortical interneurons, generating a variety of subtypes including, PV, SST, RLN, NPY, and CR interneurons as well as neurogliaform cells (Gelman et al., 2009, 2011; Niquille et al., 2018).

Lineage-tracing studies have also assessed the origin of hippocampal interneurons (Chittajallu et al., 2013; Fogarty et al., 2007; Gelman et al., 2011; Tricoire et al., 2010, 2011). Common principles as well as differences between the embryonic sources of cortical and hippocampal interneurons have been reported (reviewed in Pelkey et al., 2017). Most noticeable is the origin of *oriens lacunosum-moleculare* (O-LM) interneurons, which have their cell bodies within *stratum oriens* (*s.o.*) and represent 40% of all SST interneurons in the hippocampus (Pelkey et al., 2017). Similar to the cortex, an MGE origin for O-LM cells was initially reported using electrophysiological and morphological assessment, combined with lineage-tracing studies (Fogarty et al., 2007; Tricoire et al., 2011). A surprising second origin for ~30% of all hippocampal O-LM cells was later proposed in the CGE (Chittajallu et al., 2013). The latter conclusion was based on findings from transgenic mouse lines that label MGE and CGE interneurons, combined with physiological and functional studies (Chittajallu et al., 2013).

Recent *in situ* hybridization (ISH) mapping of *Sst* interneurons in the developing mouse brain suggested that most, if not all, telencephalic *Sst* cells originate in the diagonal area (or anterior entopeduncular area [AEP]) and not the pallidal neuroepithelium (Puelles et al., 2016). This suggestion was based on the observation that *Sst* cells appear to emerge in the mantle adjacent to the AEP, which is thought to downregulate expression of the endogenous *Nkx2-1* gene at embryonic day 13.5 (E13.5) (Puelles et al., 2016).

We previously used mice expressing Cre under control of *Lhx6* to label MGE-derived interneurons in the cortex and hippocampus (Fogarty et al., 2007). We showed that all SST-expressing interneurons in the hippocampus can be labeled in these mice and concluded an MGE origin for this population, similar to their cortical counterparts (Fogarty et al., 2007). These findings are in contrast to recent reports of a dual MGE-CGE origin for SST O-LM cells (Chittajallu et al., 2013). We therefore made use of further transgenic tools to re-address this question.

RESULTS

Genetic Lineage Tracing Shows an MGE/AEP Origin for All CA1 SST Interneurons

We used a series of Cre transgenic mice to trace the origins of hippocampal SST^{+} interneurons. Cre expression is

This is an open access article under the CC BY license (http://creativecommons.org/licenses/by/4.0/).

summarized in Figure 1A. Nkx2-1-Cre is expressed in the neuroepithelium of the MGE, AEP, and POA but lacks expression in the dorsalmost domain of the MGE (dMGE), despite robust expression of the endogenous *Nkx2-1* gene in that area (Fogarty et al., 2007). Nkx6-2-Cre is expressed in the dMGE neuroepithelium and the POA (Flames et al., 2007; Fogarty et al., 2007) and Shh-Cre is expressed in the POA (Flames et al., 2007; Flandin et al., 2010; Gelman et al., 2009). Dual transgenic mice expressing Nkx2-1-Cre and Nkx6-2-Cre label the entire MGE, AEP, and POA neuroepithelial zones (Fogarty et al., 2007). None of the mice used in this study express Cre in the LGE or the CGE (Flandin et al., 2010; Fogarty et al., 2007). We crossed Cre mice to GFP- or YFP-expressing reporter lines (Mao et al., 2001; Srinivas et al., 2001) and quantified the percentage of SST interneurons expressing GFP/YFP in *s.o.* in CA1 at postnatal day 30 (P30) (Figures 1B–1F). Around 70% of SST interneurons expressed YFP in Nkx2-1-Cre;R26R-YFP mice, indicating that the majority are generated from Nkx2-1-Cre-expressing precursors (Figures 1B and 1F). This is consistent with previous findings and suggests that the remaining 30% may be generated either outside the MGE/POA (Chittajallu et al., 2013) or from the dMGE, which does not express Cre in these mice (Figure 1A) (Fogarty et al., 2007). Analysis of Nkx6-2-Cre;R26R-GFP mice showed activation of GFP in around 30% of CA1 SST interneurons (Figures 1C and 1F), indicating either a dMGE or a POA origin for these cells. To distinguish between the two, we examined Shh-Cre;R26R-YFP mice where the POA neuroepithelium was labeled and found <5% contribution to SST interneurons (Figures 1D and 1F). This indicates that SST interneurons labeled in Nkx6-2-Cre mice originate mainly in the dMGE. The complementarity between Nkx2-1-Cre and Nkx6-2-Cre mice was confirmed in Nkx2-1-Cre;Nkx6-2-Cre;R26R-GFP triple transgenics where nearly all SST-expressing interneurons in CA1 *s.o.* were labeled with GFP (Figures 1E and 1F). Altogether, our genetic lineage-tracing analysis shows that all SST-expressing hippocampal CA1 interneurons in *s.o.* originate in *Nkx2-1*-expressing proliferative zones, as previously demonstrated for all cortical SST interneurons (Bandler et al., 2017; Kessaris et al., 2014).

The AEP Contributes to but Is Not the Sole Source of Telencephalic SST Interneurons

Recent ISH mapping of *Sst* interneurons in the developing mouse brain suggested that most, if not all, telencephalic *Sst* cells originate in the AEP (or diagonal area) and that failure to label 30% of cortical interneurons in Nkx2-1-Cre mice may have been caused by lack of NKX2-1 expression in the AEP at E13.5 (Puelles et al., 2016). We could not detect downregulation of NKX2-1 in the AEP at E13.5 (Figure S1A) and, in contrast to the dMGE, which fails to activate expression of YFP in our Nkx2-1-Cre;R26R-YFP mice (Figure S1A) (Fogarty et al., 2007), we find robust activation of the Rosa26R-YFP allele in the AEP at E13.5 (Figure S1A). This indicates that the 30% of SST interneurons that failed to be labeled in the Nkx2-1-Cre; R26R-YFP mouse are not generated from the AEP. Instead, in Nkx6-2-Cre mice, which express Cre in the dMGE but not in the AEP (Figure S1B) (Fogarty et al., 2007), 30% of hippocampal SST interneurons are labeled and complement the Nkx2-1-Cre mice (Figure 1F). Hence, we can conclude that the AEP may contribute to but is not the sole or principal source of SST interneurons, and it is not the source of 30% of hippocampal SST interneurons that are unlabeled in our Nkx2-1-Cre mice.

SST-Expressing Cells Are Generated in the Absence of NKX2-1

To clarify the requirement for NKX2-1 in the generation of SST interneurons, we examined *Nkx2-1* germline knockout (KO) embryos for the presence of SST-positive cells. At E13.5, most *Sst* cells were missing from the telencephalon in *Nkx2-1* KO embryos compared with controls (Figures 2A and 2B). However, a clear stream of *Sst* cells seemingly emerging from the AEP could be detected in mutant embryos (Figure 2B). Activation of *Lhx6*, a direct transcriptional target of NKX2-1 (Du et al., 2008; Sandberg et al., 2016), can be detected in mutant embryos near the *Sst*$^{+}$ zone, but expression is not maintained in migrating SST cells (Figures 2C and 2D). At E18.5, the latest stage at which these embryos can be examined due to postnatal lethality, we could detect *Sst*-expressing cells in subcortical regions such as the developing amygdala, but these did not express *Lhx6* (Figures 2E–2H). In contrast, the hippocampus was devoid of *Sst*- and *Lhx6*-expressing cells (Figures 2I–2L). Altogether, our data indicate that subcortical *Sst*$^{+}$ cells can be generated in the absence of *Nkx2-1*, but these do not maintain expression of *Lhx6* and do not migrate to the hippocampus. Whether these represent an *Nkx2-1/Lhx6*-independent *Sst* population or an abnormal population of *Sst* cells generated in these mutants remains unknown.

A Single LHX6 Origin for All Hippocampal SST Interneurons

We previously showed that nearly 100% of hippocampal CA1 SST interneurons are labeled with YFP in Lhx6-Cre;R26R-YFP transgenic mice (Fogarty et al., 2007). We extended this work by assessing SST interneurons at all anterior-posterior levels of CA1. We confirmed a near-complete co-localization of SST with YFP (Figures 3A–3C). To determine whether SST interneurons maintain LHX6 expression in postnatal animals, we used transgenic

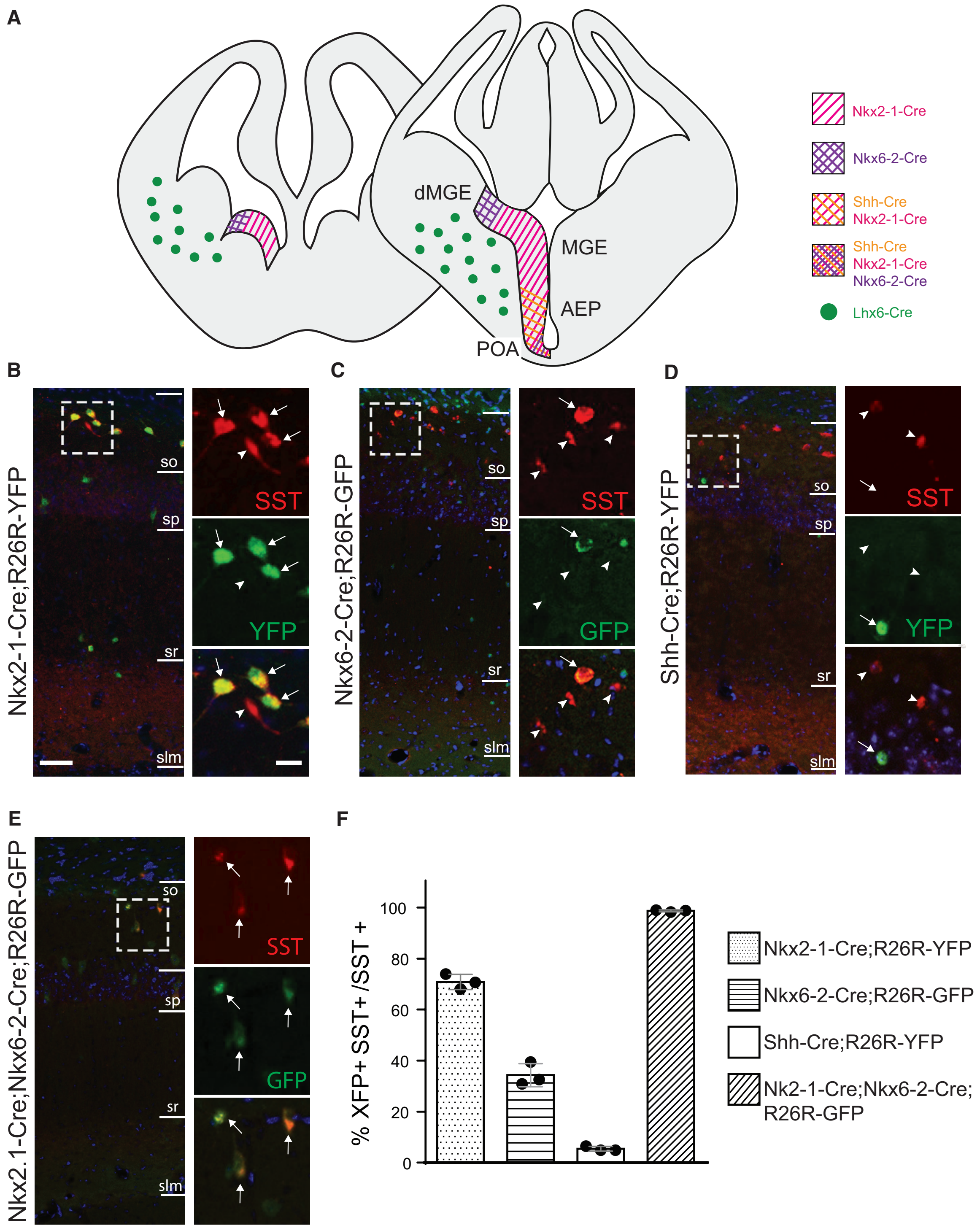

Figure 1. Genetic Lineage Tracing of SST Interneurons in CA1

(A) Cre expression in the ventral telencephalon in transgenic mice used in this study.

(B–E) Immunohistochemistry detecting CA1 SST interneurons expressing XFP in Nkx2-1-Cre;R26R-YFP (B), Nkx6-2-Cre;R26R-GFP (C), Shh-Cre;R26R-YFP (D), and Nkx2-1-Cre;Nkx6-2-Cre;R26R-GFP (E) at P30. Boxed areas are shown at higher magnification. Arrows show double-positive cells and arrowheads show SST^{+}/XFP^{-} cells.

(F) Quantification of XFP/SST interneurons as a percentage of SST cells in CA1 *stratum oriens* (*s.o.*) at P30. Mean ± SD.

Scale bars, 20 μm (left images) and 5 μm (right images).

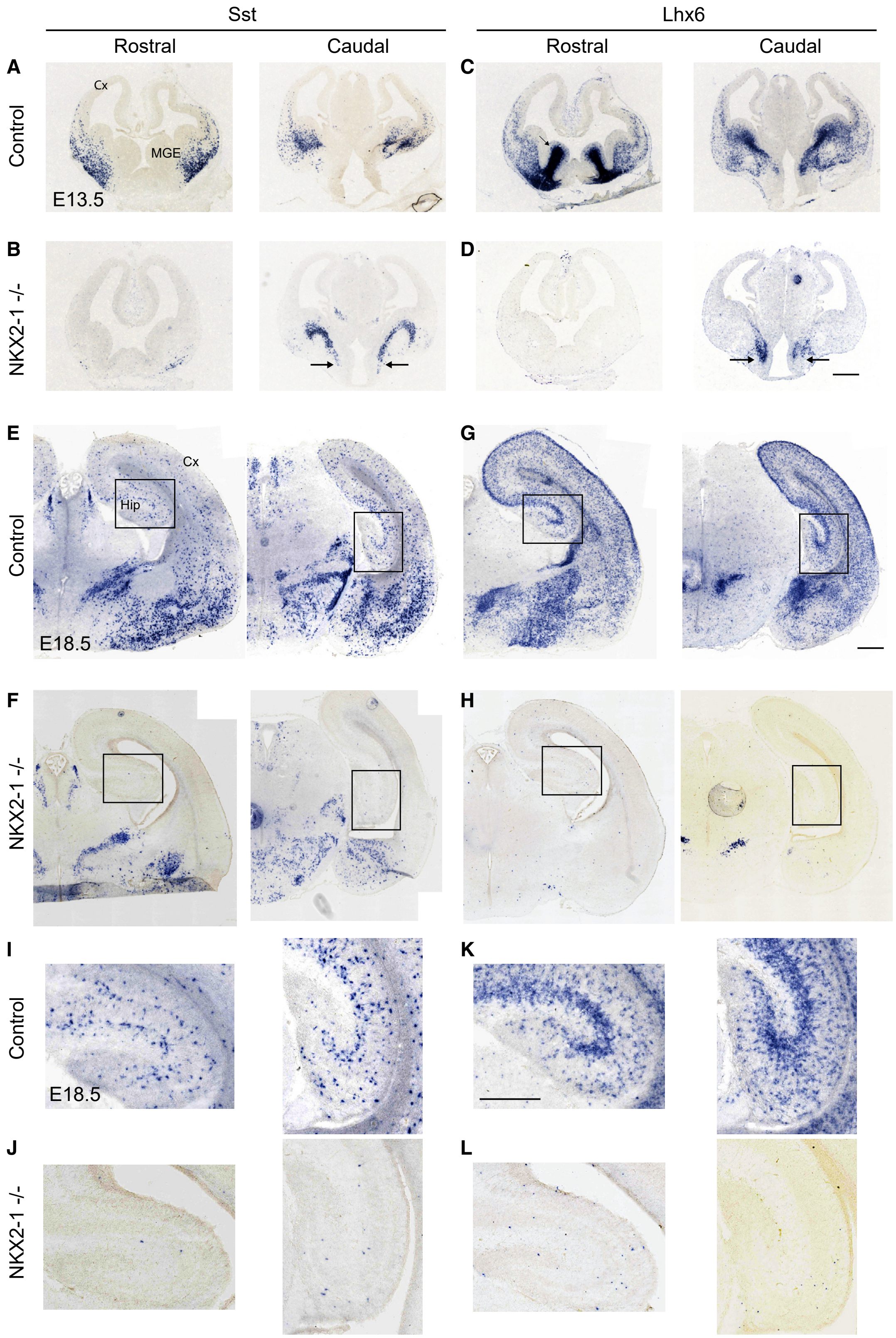

(legend on next page)

mice expressing Cre under control of *Sst* (Taniguchi et al., 2011). Immunohistochemistry for LHX6 and YFP and quantification of co-localization between the two in CA1 *s.o.* showed that the majority of SST CA1 interneurons maintain LHX6 expression in the adult (Figures 3D and 3E). However, there was a marked variability in the levels of expression of LHX6, and a small proportion of YFP cells appeared negative for LHX6 (~15%) (Figures 3D and 3E). This variability was also observed in the cortex, although a smaller proportion was negative for LHX6 in this region (~5%) (Figures 3D and 3F). These LHX6-negative/YFP-positive cells in Sst-Cre;R26R-YFP transgenic mice may simply express very low levels of LHX6, undetectable with our protocols. Alternatively, they may represent non-SST cells, ectopically expressing Cre, as recently suggested (Hu et al., 2013; Mikulovic et al., 2015). Altogether, the data indicate that SST CA1 interneurons in *s.o.* originate from LHX6-expressing progenitors and the vast majority maintain LHX6 expression at adult stages.

Single-Cell Transcriptomic Analysis Shows Clustering of Hippocampal *Sst*/*Htr3a* Interneurons with MGE Populations

To detect the presence of *Htr3a*-expressing *Sst* interneurons in the adult hippocampus, we took advantage of our recent RNA transcriptomics dataset of 3,283 interneurons from CA1 (Harris et al., 2018). Based on their transcriptomic profiles, CA1 interneurons could be subdivided into 49 major clusters that can be arranged in 10 "continents" on an nbtSNE map (Harris et al., 2018). Clusters originating in the MGE can be identified in "continents" 1, 2, 3, 4, and 10 (indicated in Figure 4A) (Harris et al., 2018). High *Htr3a* expression is observed in CGE-derived clusters, which do not express *Sst* (Figures 4A and 4B). Among MGE populations, low expression of *Htr3a* is observed in a few clusters in continents 1 and 10, which co-express *Sst* (circled in red in Figure 4A). These include putative O-LM and hippocamposeptal neurons. Our data demonstrate that *Sst* and *Htr3a*-co-expressing cells are present in small numbers in CA1, although their expression profile is more similar to MGE rather than CGE-derived interneurons.

DISCUSSION

We used a range of genetic lineage-tracing mice together with single-cell transcriptomic analysis to re-examine the origin of hippocampal CA1 *Sst*-expressing interneurons. We find that all *Sst* cells in CA1 are generated from *Nkx2-1*-expressing neuroepithelial cells and can be labeled with Lhx6-Cre transgenic mice. Low expression of *Htr3a* can be detected in some *Sst* CA1 interneurons but their transcriptomic signature clusters them together with MGE-derived populations. Altogether, our data indicate a single MGE/POA origin for all hippocampal CA1 SST interneurons.

A dual MGE-CGE origin for CA1 O-LM cells had recently been suggested (Chittajallu et al., 2013). While an MGE origin parallels that of SST interneurons in the cortex, the finding of a CGE origin for *Htr3a*$^+$*Sst*$^+$ O-LM cells was surprising. Apart from expression of 5-HT$_{3A}$R and consequent preferential response to serotonin, MGE and CGE O-LM cells were found to have identical characteristics. These included laminar position, neurochemical content, basic membrane and spiking properties, as well as muscarinic and mGluR1 or mGluR5 response profiles (Chittajallu et al., 2013). Three observations led to the suggestion that SST$^+$5-HT$_{3A}$R$^+$ O-LM cells have a CGE origin: (1) ~30% of SST cells with typical O-LM characteristics were labeled with GFP in Htr3a-GFP mice; (2) a proportion of *s.o.* SST O-LM cells were labeled with GFP in Mash1-CreER;RCE transgenic mice following tamoxifen administration; and (3) there was only ~10% overlap in expression of tdTomato and GFP in Nkx2-1-Cre;tdTOM;Htr3a-GFP mice (Chittajallu et al., 2013). Together, these observations suggested a CGE origin for ~30% of SST O-LM cells. However, there are known caveats to the transgenic mice used: Nkx2-1-Cre mice fail to express Cre in the dMGE, resulting in large numbers of unlabeled MGE cells in lineage-tracing experiments (Xu et al., 2008); *Mash1* is expressed throughout the MGE and CGE neuroepithelium (Casarosa et al., 1999), and the extent of preferential CGE labeling depends on the dose and time of tamoxifen administration or even the genetic background of the mice used. In Htr3a-Cre;GFP and Htr3a-GFP mice, labeled cells can be seen to emanate not only from the CGE but also from the

Figure 2. Expression of *Sst* and *Lhx6* in Control and Mutant Embryos Lacking NKX2-1
(A–D) E13.5 coronal sections showing expression of *Sst* (A and B) and *Lhx6* (C and D). Most *Sst* expression is missing from the telencephalon of Nkx2-1$^{-/-}$ embryos compared with controls. A clear stream of *Sst* cells appears to be emerging from the AEP in mutants (arrows in B) but these do not appear to migrate to the cortex. *Lhx6* can only be detected near the AEP in mutant embryos (arrows in D). Expression is not maintained in migrating cells.
(E–H) E18.5 coronal sections showing expression of *Sst* (E and F) and *Lhx6* (G and H). *Sst* cells can be detected in the subcortical telencephalon in *Nkx2-1* mutant embryos and these do not express *Lhx6*. Boxed areas are shown in high magnification in (I) to (L).
(I–L) The hippocampus is devoid of *Sst* and *Lhx6* expression in *Nkx2-1* mutant embryos.
Scale bars, 200 µm (A–H) and 150 µm (I–L).

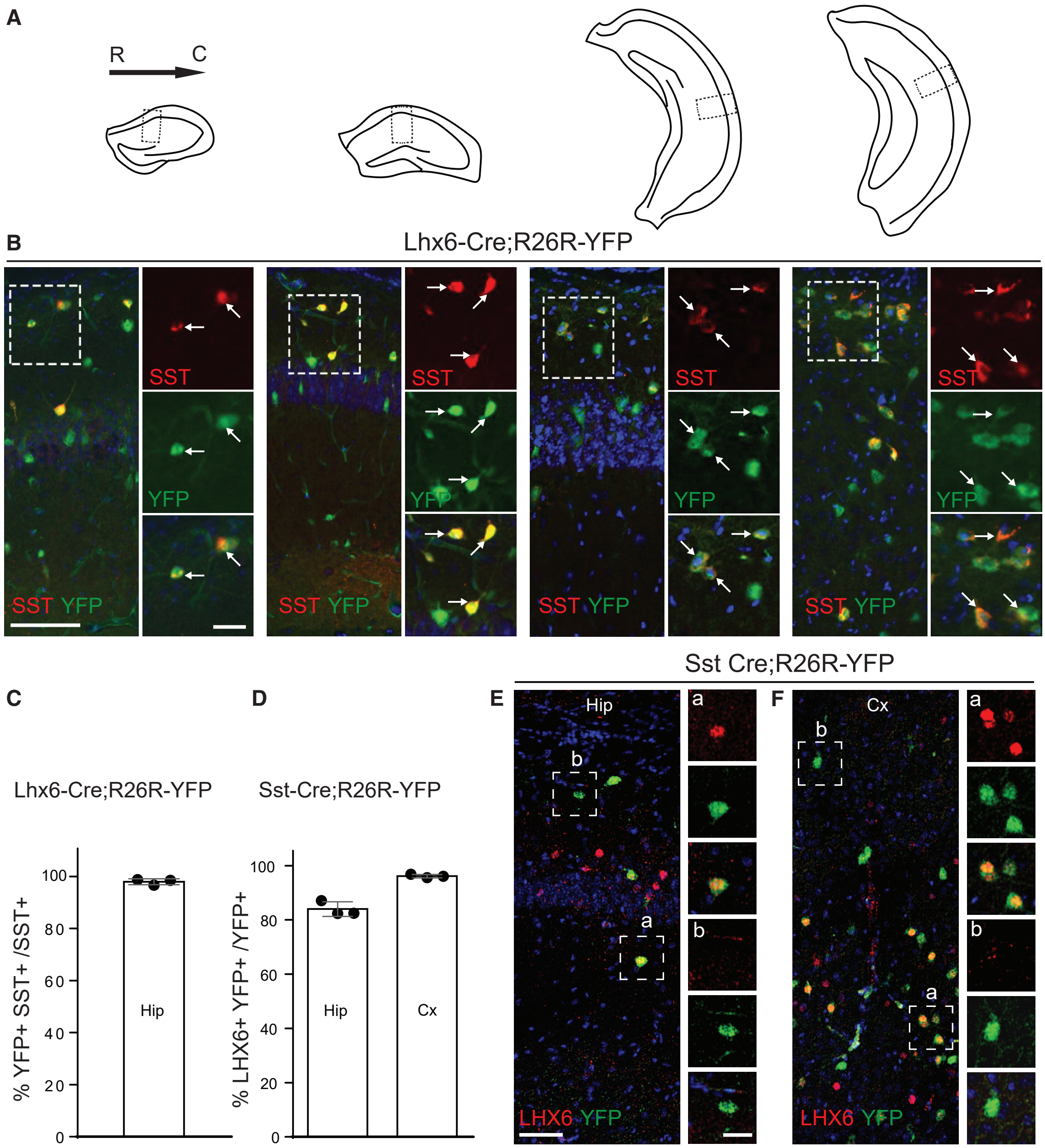

Figure 3. Genetic Labeling of SST CA1 Interneurons in Adult Lhx6-Cre;R26R-YFP Transgenic Mice and Expression of LHX6 in YFP Cells in Sst-Cre;R26-YFP Mice

(A) Schematics showing the different rostrocaudal levels of the hippocampus examined in (B).

(B) Co-expression of YFP with SST in Lhx6-Cre;R26R-YFP mice at P30 at different rostrocaudal levels of CA1. Boxed areas are shown at higher magnification. Arrows point to double-labeled cells.

(C) Quantification of YFP/SST interneurons as a percentage of SST cells in CA1 *s.o.* at P30 in Lhx6-Cre;R26R-YFP mice. Mean ± SD.

(D) Quantification of YFP/LHX6 interneurons as a percentage of YFP cells in the hippocampus (Hip) CA1 *s.o.* and the cortex (Cx) in SST-Cre;R26R-YFP mice at P30. Mean ± SD.

(E and F) Co-expression of YFP with SST in the hippocampus (hip) CA1 *s.o.* (E) and the cortex (Cx) (F) in Sst-Cre;R26R-YFP mice at P30. Boxed areas are shown at higher magnification. Scale bars in (B), 30 μm (left) and 50 μm (right); scale bars in (E), 50 μm (left) and 15 μm (right).

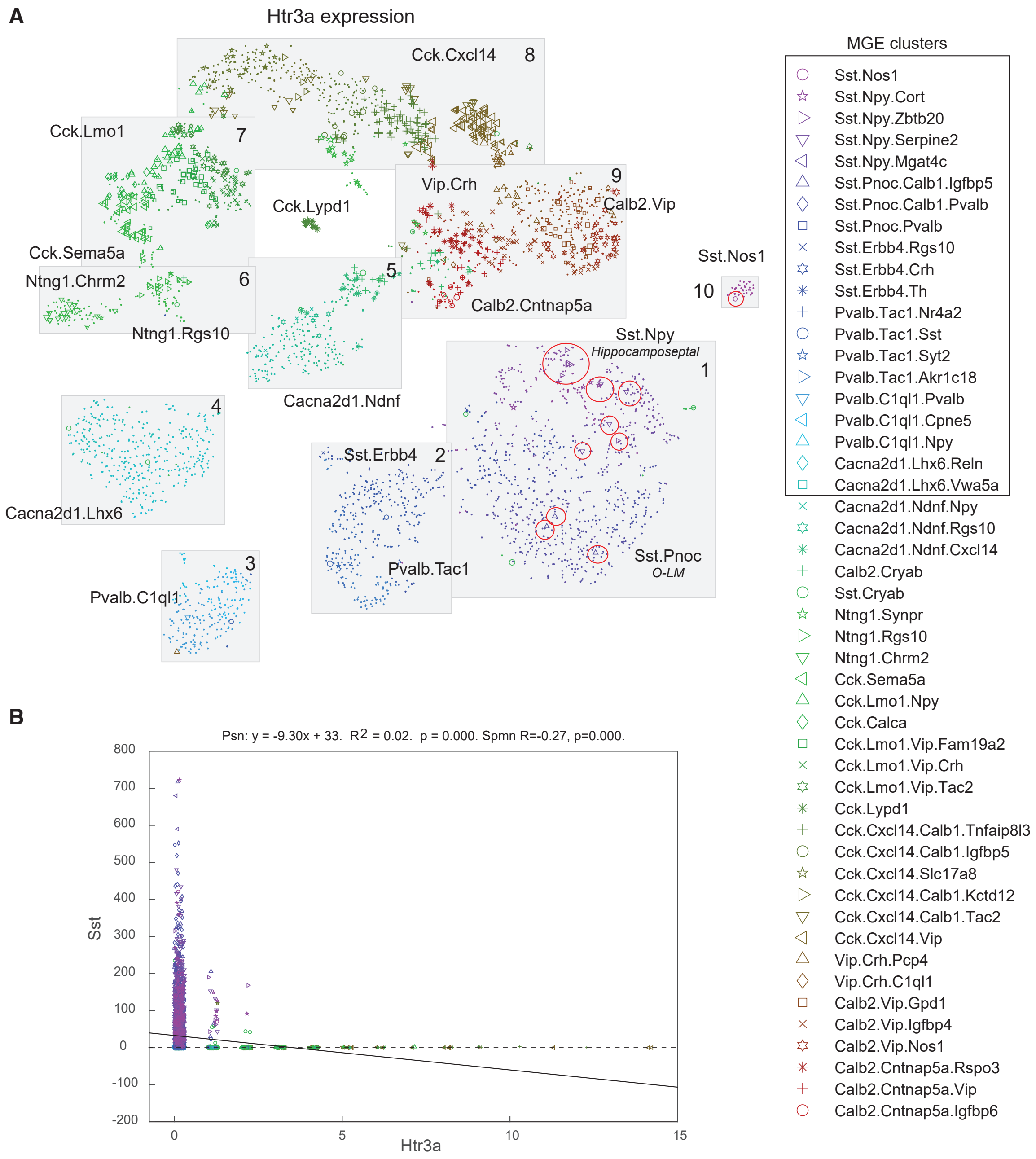

Figure 4. Single-Cell Transcriptomic Analysis Shows Clustering of Hippocampal *Sst/Htr3a* Interneurons with MGE Populations

(A) Expression levels of *Htr3a* on an nbtSNE map of all CA1 interneuron clusters. Boxes refer to cell clusters previously described in Harris et al. (2018). Each symbol represents a cell type that belongs to a specific group (legend, right). The size of the symbol on the map shows the level of *Htr3a* expression (log-expression). There are only a few *Sst/Htr3a* co-expressing cells and these are grouped with previously described MGE clusters (red circles).

(B) Scatterplot showing the correlation between expression of *Sst* and *Htr3a* over all 3,283 CA1 interneurons in the database. A few *Sst* cells express low levels of *Htr3a*. There is no significant linear correlation between the expression of *Htr3a* (x axis) and *Sst* (y axis) in these cells. Solid line: correlation coefficient. Dashed line: x = 0.

AEP/POA, and hence cannot be used to exclusively trace the CGE (Vucurovic et al., 2010). Finally, as *Htr3a* is a post-mitotic marker, it is difficult to exclude the possibility of upregulation of the gene during migration or at any later stage during the maturation of these cells, independently of the neuroepithelial origin of these cells.

In the present study, we used transgenic mice expressing Cre in neuroepithelial cells and showed that all SST CA1 interneurons are generated in the MGE/POA, including the dMGE, which fails to express Cre in Nkx2-1Cre mice (Fogarty et al., 2007). Lhx6-Cre;Rosa26YFP mice also label all SST interneurons in CA1 with YFP. Even though *Lhx6* does not delineate a neuroepithelial domain, it is a direct target of NKX2-1 and hence labels postmitotic neurons generated from *Nkx2-1*-expressing precursors (Du et al., 2008; Fogarty et al., 2007; Sandberg et al., 2016). To further examine the possibility that some SST cells may be generated from the CGE, we mined our single-cell transcriptomic data (Harris et al., 2018). *Sst*-expressing cells clustered together on nbtSNE maps based on their transcriptomic profile, and these were clearly separate from CGE clusters. Cells expressing low levels of Htr3a were found among SST clusters but their profiles were more similar to MGE populations than CGE ones. Altogether, our data point toward an MGE/POA origin for all hippocampal CA1 SST interneurons.

Interestingly, we found that some SST-expressing cells are generated in the absence of NKX2-1 in the AEP/POA regions. These cells remain within the subcortical telencephalon and do not migrate to the cortex or the hippocampus. It is possible that other NKX genes, such as *Nkx2-4* or *Nkx6-2*, may compensate for the loss of NKX2-1 in AEP/POA regions in NKX2-1 mutants, thereby allowing the generation of SST interneurons in the absence of NKX2-1. For example, nkx2-4 is expressed in the hypothalamus and is functionally redundant with nkx2-1 in zebrafish (Manoli and Driever, 2014). Alternatively, some SST cells may normally be generated in the AEP/POA regions independently of NKX2-1, but these do not migrate to the cortex or the hippocampus.

Knowledge of the embryonic origin of cortical GABAergic interneurons and genetic programs that drive distinct interneuron fates has been instrumental in the generation of these cells *in vitro* for putative stem cell-based therapeutic approaches (Tyson and Anderson, 2014). Much of this knowledge has come from genetic lineage tracing in transgenic mice using recombinases. However, these come with caveats of their own: mice expressing Cre in neuroepithelial domains need to be well characterized spatially and temporally, not only by expression of the endogenous gene but also of the Cre transgene and the activation of the Cre reporter gene. This is because the Cre transgene may not fully recapitulate the expression of the endogenous gene. In addition, parameters such as accessibility of *loxP* sites and distance between them will determine the ease with which recombination takes place, thereby masking or unmasking low levels of expression of Cre and causing variable reporting depending on the model used. Even more caution should be exercised when carrying out lineage-tracing experiments based on mice expressing a reporter gene such as GFP or other fluorescent proteins in postmitotic neurons because of the difficulty in tracing the history of activation of that promoter throughout embryogenesis and into adulthood. Single-cell transcriptomics is now transforming developmental and evolutionary biology by providing us with unprecedented insight into the transcriptomic makeup of single cells. However, the technique inherently lacks positional information and, being relatively new, is still rife with experimental and computational caveats. Combining genetic lineage tracing and single-cell transcriptomics provides us with a powerful method for identifying developmental cell diversifications and deciphering *in vivo* cell lineages, information that forms the basis for stem cell studies and the generation of differentiated cells *in vitro*.

EXPERIMENTAL PROCEDURES

Animals

$Tg^{(Nkx2\text{-}1\text{-}cre)1Wdr}$ (MGI:3761164) (Kessaris et al., 2006), $Tg^{(Nkx6\text{-}2\text{-}icre)1Kess}$ (MGI:4355562) (JAX 027798), $Tg^{(Lhx6\text{-}icre)1Kess}$ (MGI:4355717) (JAX 026555) (Fogarty et al., 2007), $Shh^{tm1(EGFP/cre)Cjt}$ (MGI:3053959) (JAX 005622) (Harfe et al., 2004), $Sst^{tm2.1(cre)Zjh}$ (MGI:4838416) (JAX 013044) (Taniguchi et al., 2011), R26R-YFP^{KI} (JAX 006148) (Srinivas et al., 2001), and R26R-GFP^{KI} reporter mice (JAX 004077) (Mao et al., 2001) have been described previously. We refer to them herein as Nkx2-1-Cre, Nkx6-2-Cre, Lhx6-Cre, Shh-Cre, Sst-Cre, R26R-YFP, and R26R-GFP, respectively. Mice carrying a germline deletion in *Nkx2-1* were generated by germline recombination of a floxed allele (Kusakabe et al., 2006). All animals used in this study were maintained on a mixed C57BL6/CBA background at the Wolfson Institute for Biomedical Research, University College of London in accordance with United Kingdom legislation (ASPA 1986).

Tissue Processing, Immunohistochemistry, and ISH

Tissue processing, immunohistochemistry, and ISH were carried out as previously described (Magno et al., 2012; Rubin et al., 2010). Primary antibodies used were: rat monoclonal anti-GFP immunoglobulin G (IgG) 2a (GF090R) (1:1,000, Nacalai Tesque, cat. no. 04404-84), rabbit polyclonal anti-SST (1:200, Peninsula Labs, cat. no. T-4103.0050), rabbit polyclonal anti-TTF-1 IgG (NKX2-1) (1:100, Santa Cruz Biotechnology, cat. no. sc-13040), and rabbit polyclonal anti-LHX6 (a gift from V. Pachnis). Alexa-Fluor conjugated secondary antibodies were used at 1:1,000 (Thermo Fisher) (donkey anti-rabbit 568, cat. no. A-10042, donkey anti-rat 488, cat. no. A-21208). For RNA ISH, *Lhx6*- and *Sst* DIG-labeled probes were generated from a plasmid (kind gift from V. Pachnis) and IMAGE clone 4218815 (Source Biosciences), respectively.

Imaging and Quantification

Images were captured using a Hamamatsu C4742-95 camera on a Zeiss Axioplan fluorescence microscope and Digital Pixel

software. Confocal images were captured on a Leica CTR6500 confocal microscope or a Zeiss LSM880 with Airyscan. ISH images were captured on a Zeiss Axio Scan.Z1 scanner. Image composites were assembled using Microsoft ICE software (Microsoft, Redmond, WA) and processed with Adobe Photoshop CS6 (Adobe Systems, San Jose, CA). Figures were generated in Adobe Illustrator CS6 (Adobe Systems). Quantification was performed as described previously (Fogarty et al., 2007; Magno et al., 2012). Three animals were used in each experiment and quantification was performed on 4–6 hippocampal CA1 regions from 2–3 sections per animal (30 μm thickness). A total of 400–600 SST^+ cells per animal were counted in order to generate the data shown in Figures 1 and 3. Two-tailed t tests were used with an alpha of 0.05.

Single-Cell RNA-Sequencing Analysis

The single-cell data presented were obtained by reanalyzing our previously published datasets (Harris et al., 2018). The code for cluster analysis and all other algorithms can be found at https://github.com/cortexlab/Transcriptomics. To visualize different cell subtypes, we used the negative binomial t-stochastic neighbor-embedding (nbtSNE) algorithm as previously described (Harris et al., 2018).

SUPPLEMENTAL INFORMATION

Supplemental Information can be found online at https://doi.org/10.1016/j.stemcr.2019.09.008.

AUTHOR CONTRIBUTIONS

Conceptualization, Z.A. and N. Kessaris; Methodology, N. Kessaris and K.D.H.; Investigation, Z.A., L.M., N. Ktena, K.D.H., and N. Kessaris; Writing – Original Draft, Z.A. and N. Kessaris; Writing – Review & Editing, Z.A., L.M., K.D.H., and N. Kessaris; Funding Acquisition, K.D.H. and N. Kessaris.

ACKNOWLEDGMENTS

We thank our colleagues at the Wolfson Institute for Biomedical Research (University College London) for helpful comments and discussions. Financial support for the work was provided by the Wellcome Trust (108726/Z/15/Z) to K.D.H. and N Kessaris, and the UK Biotechnology and Biological Sciences Research Council (BB/N009061/1) to N. Kessaris.

Received: June 4, 2019
Revised: September 19, 2019
Accepted: September 20, 2019
Published: October 17, 2019

REFERENCES

Bandler, R.C., Mayer, C., and Fishell, G. (2017). Cortical interneuron specification: the juncture of genes, time and geometry. Curr. Opin. Neurobiol. *42*, 17–24.

Casarosa, S., Fode, C., and Guillemot, F. (1999). Mash1 regulates neurogenesis in the ventral telencephalon. Development *126*, 525–534.

Chittajallu, R., Craig, M.T., McFarland, A., Yuan, X., Gerfen, S., Tricoire, L., Erkkila, B., Barron, S.C., Lopez, C.M., Liang, B.J., et al. (2013). Dual origins of functionally distinct O-LM interneurons revealed by differential 5-HTR expression. Nat. Neurosci. *16*, 1598–1607.

Du, T., Xu, Q., Ocbina, P.J., and Anderson, S.A. (2008). NKX2.1 specifies cortical interneuron fate by activating Lhx6. Development *135*, 1559–1567.

Flames, N., Pla, R., Gelman, D.M., Rubenstein, J.L., Puelles, L., and Marin, O. (2007). Delineation of multiple subpallial progenitor domains by the combinatorial expression of transcriptional codes. J. Neurosci. *27*, 9682–9695.

Flandin, P., Kimura, S., and Rubenstein, J.L. (2010). The progenitor zone of the ventral medial ganglionic eminence requires Nkx2-1 to generate most of the globus pallidus but few neocortical interneurons. J. Neurosci. *30*, 2812–2823.

Fogarty, M., Grist, M., Gelman, D., Marin, O., Pachnis, V., and Kessaris, N. (2007). Spatial genetic patterning of the embryonic neuroepithelium generates GABAergic interneuron diversity in the adult cortex. J. Neurosci. *27*, 10935–10946.

Gelman, D., Griveau, A., Dehorter, N., Teissier, A., Varela, C., Pla, R., Pierani, A., and Marin, O. (2011). A wide diversity of cortical GABAergic interneurons derives from the embryonic preoptic area. J. Neurosci. *31*, 16570–16580.

Gelman, D.M., Martini, F.J., Nobrega-Pereira, S., Pierani, A., Kessaris, N., and Marin, O. (2009). The embryonic preoptic area is a novel source of cortical GABAergic interneurons. J. Neurosci. *29*, 9380–9389.

Harfe, B.D., Scherz, P.J., Nissim, S., Tian, H., McMahon, A.P., and Tabin, C.J. (2004). Evidence for an expansion-based temporal Shh gradient in specifying vertebrate digit identities. Cell *118*, 517–528.

Harris, K.D., Hochgerner, H., Skene, N.G., Magno, L., Katona, L., Bengtsson Gonzales, C., Somogyi, P., Kessaris, N., Linnarsson, S., and Hjerling-Leffler, J. (2018). Classes and continua of hippocampal CA1 inhibitory neurons revealed by single-cell transcriptomics. Plos Biol. *16*, e2006387.

Hu, H., Cavendish, J.Z., and Agmon, A. (2013). Not all that glitters is gold: off-target recombination in the somatostatin-IRES-Cre mouse line labels a subset of fast-spiking interneurons. Front. Neural Circuits *7*, 195.

Kessaris, N., Fogarty, M., Iannarelli, P., Grist, M., Wegner, M., and Richardson, W.D. (2006). Competing waves of oligodendrocytes in the forebrain and postnatal elimination of an embryonic lineage. Nat. Neurosci. *9*, 173–179.

Kessaris, N., Magno, L., Rubin, A.N., and Oliveira, M.G. (2014). Genetic programs controlling cortical interneuron fate. Curr. Opin. Neurobiol. *26C*, 79–87.

Kusakabe, T., Kawaguchi, A., Hoshi, N., Kawaguchi, R., Hoshi, S., and Kimura, S. (2006). Thyroid-specific enhancer-binding protein/NKX2.1 is required for the maintenance of ordered architecture and function of the differentiated thyroid. Mol. Endocrinol. *20*, 1796–1809.

Magno, L., Oliveira, M.G., Mucha, M., Rubin, A.N., and Kessaris, N. (2012). Multiple embryonic origins of nitric oxide synthase-expressing GABAergic neurons of the neocortex. Front. Neural Circuits *6*, 65.

Manoli, M., and Driever, W. (2014). nkx2.1 and nkx2.4 genes function partially redundant during development of the zebrafish hypothalamus, preoptic region, and pallidum. Front. Neuroanat. *8*, 145.

Mao, X., Fujiwara, Y., Chapdelaine, A., Yang, H., and Orkin, S.H. (2001). Activation of EGFP expression by Cre-mediated excision in a new ROSA26 reporter mouse strain. Blood *97*, 324–326.

Mikulovic, S., Restrepo, C.E., Hilscher, M.M., Kullander, K., and Leao, R.N. (2015). Novel markers for OLM interneurons in the hippocampus. Front. Cell Neurosci. *9*, 201.

Miyoshi, G., Hjerling-Leffler, J., Karayannis, T., Sousa, V.H., Butt, S.J., Battiste, J., Johnson, J.E., Machold, R.P., and Fishell, G. (2010). Genetic fate mapping reveals that the caudal ganglionic eminence produces a large and diverse population of superficial cortical interneurons. J. Neurosci. *30*, 1582–1594.

Niquille, M., Limoni, G., Markopoulos, F., Cadilhac, C., Prados, J., Holtmaat, A., and Dayer, A. (2018). Neurogliaform cortical interneurons derive from cells in the preoptic area. eLife *7*. https://doi.org/10.7554/eLife.32017.

Pelkey, K.A., Chittajallu, R., Craig, M.T., Tricoire, L., Wester, J.C., and McBain, C.J. (2017). Hippocampal GABAergic inhibitory interneurons. Physiol. Rev. *97*, 1619–1747.

Puelles, L., Morales-Delgado, N., Merchan, P., Castro-Robles, B., MartInez-de-la-Torre, M., Diaz, C., and Ferran, J.L. (2016). Radial and tangential migration of telencephalic somatostatin neurons originated from the mouse diagonal area. Brain Struct. Funct. *221*, 3027–3065.

Rubin, A.N., Alfonsi, F., Humphreys, M.P., Choi, C.K., Rocha, S.F., and Kessaris, N. (2010). The germinal zones of the basal ganglia but not the septum generate GABAergic interneurons for the cortex. J. Neurosci. *30*, 12050–12062.

Sandberg, M., Flandin, P., Silberberg, S., Su-Feher, L., Price, J.D., Hu, J.S., Kim, C., Visel, A., Nord, A.S., and Rubenstein, J.L. (2016). Transcriptional networks controlled by NKX2-1 in the development of forebrain GABAergic neurons. Neuron *91*, 1260–1275.

Srinivas, S., Watanabe, T., Lin, C.S., William, C.M., Tanabe, Y., Jessell, T.M., and Costantini, F. (2001). Cre reporter strains produced by targeted insertion of EYFP and ECFP into the ROSA26 locus. BMC Dev. Biol. *1*, 4.

Taniguchi, H., He, M., Wu, P., Kim, S., Paik, R., Sugino, K., Kvitsiani, D., Fu, Y., Lu, J., Lin, Y., et al. (2011). A resource of Cre driver lines for genetic targeting of GABAergic neurons in cerebral cortex. Neuron *71*, 995–1013.

Tricoire, L., Pelkey, K.A., Daw, M.I., Sousa, V.H., Miyoshi, G., Jeffries, B., Cauli, B., Fishell, G., and McBain, C.J. (2010). Common origins of hippocampal Ivy and nitric oxide synthase expressing neurogliaform cells. J. Neurosci. *30*, 2165–2176.

Tricoire, L., Pelkey, K.A., Erkkila, B.E., Jeffries, B.W., Yuan, X., and McBain, C.J. (2011). A blueprint for the spatiotemporal origins of mouse hippocampal interneuron diversity. J. Neurosci. *31*, 10948–10970.

Tyson, J.A., and Anderson, S.A. (2014). GABAergic interneuron transplants to study development and treat disease. Trends Neurosci. *37*, 169–177.

Vucurovic, K., Gallopin, T., Ferezou, I., Rancillac, A., Chameau, P., van Hooft, J.A., Geoffroy, H., Monyer, H., Rossier, J., and Vitalis, T. (2010). Serotonin 3A receptor subtype as an early and protracted marker of cortical interneuron subpopulations. Cereb. Cortex *20*, 2333–2347.

Wonders, C.P., and Anderson, S.A. (2006). The origin and specification of cortical interneurons. Nat. Rev. Neurosci. *7*, 687–696.

Xu, Q., Tam, M., and Anderson, S.A. (2008). Fate mapping Nkx2.1-lineage cells in the mouse telencephalon. J. Comp. Neurol. *506*, 16–29.

Stem Cell Reports, Volume *13*

Supplemental Information

Hippocampal CA1 Somatostatin Interneurons Originate in the Embryonic MGE/POA

Zeinab Asgarian, Lorenza Magno, Niki Ktena, Kenneth D. Harris, and Nicoletta Kessaris

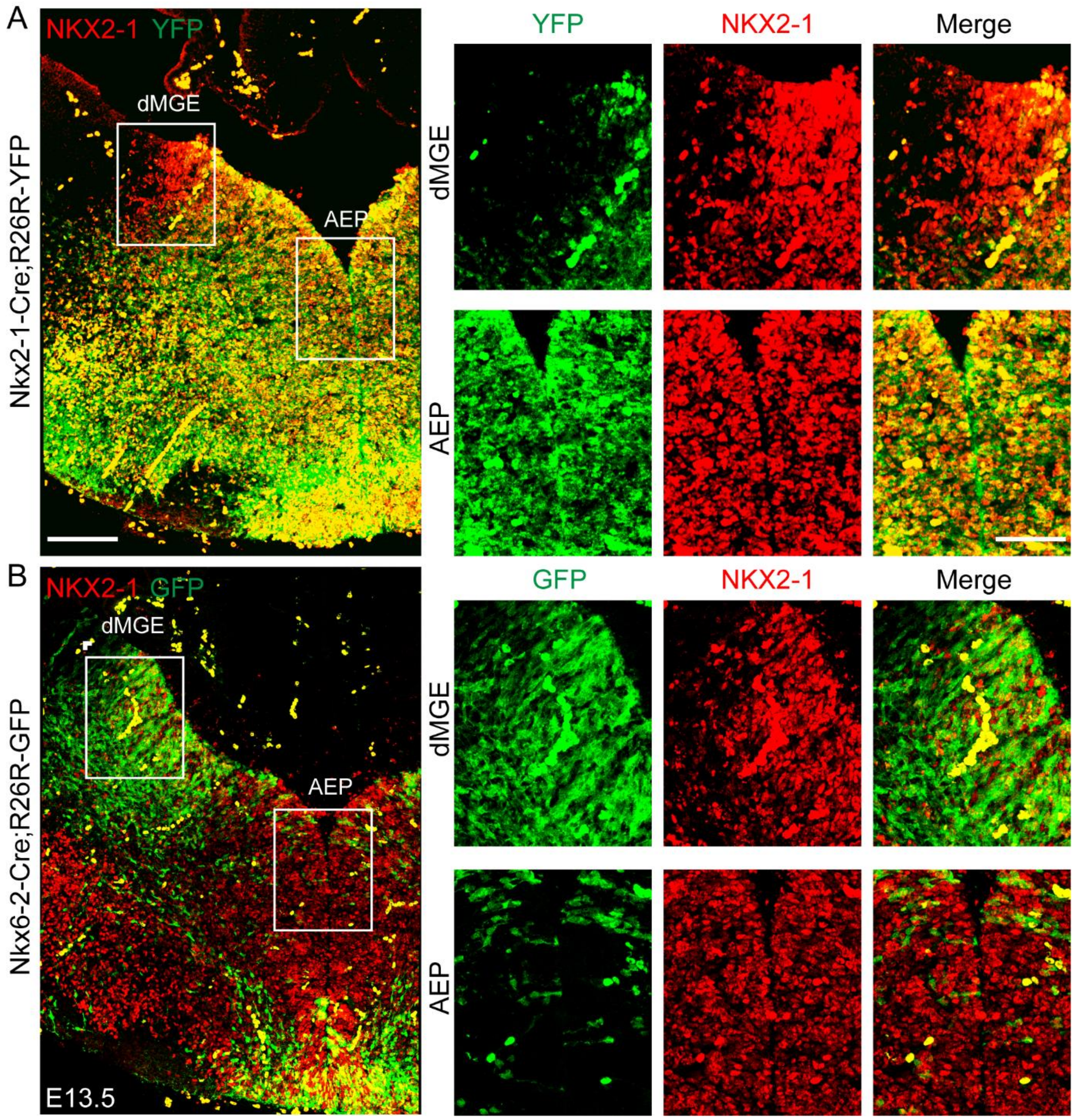

Figure S1 (Related to Figure 1):

Expression of NKX2-1 protein in the MGE and AEP in Nkx2-1-Cre;YFP and Nkx6-2-Cre;GFP mice at E13.5

A) NKX2-1 expression in Nkx2-1-Cre;YFP at E13.5. Expression of endogenous NKX2-1 and activation of YFP expression overlap in the MGE and AEP but not in the dMGE. The dMGE and AEP are shown at higher magnification.

B) NKX2-1 expression in Nkx6-2-Cre;GFP at E13.5. Activation of GFP expression is observed in the dMGE where NKX2-1 protein can be detected but not in the AEP. dMGE, dorsal medial ganglionic eminence; AEP, anterior entopeduncular area. Scale bars: a, b: 100 μm (left), 60 μm (right).
